# New-onset or flare-up of bullous pemphigoid associated with COVID-19 vaccines: a systematic review of case report and case series studies

**DOI:** 10.3389/fmed.2024.1293920

**Published:** 2024-04-08

**Authors:** Aref Ghanaatpisheh, Mohadesseh Safari, Hoda Haghshenas, Ali Motamed-Sanaye, Amir Homayoun Atefi, Karo Kamangarpour, Mohammad Aref Bagherzadeh, Amirhossein Kamran-Jahromi, Mohammad Darayesh, Navid Kouhro, Amir Reza Bahadori, Mohammad Ali Esfandiari

**Affiliations:** ^1^Student Research Committee, Jahrom University of Medical Sciences, Jahrom, Iran; ^2^Student Research Committee, School of Medicine, Guilan University of Medical Sciences, Rasht, Iran; ^3^Infectious Diseases Research Center, Student Research Committee, Faculty of Medicine, Gonabad University of Medical Sciences, Gonabad, Iran; ^4^Social Determinants of Health Research Center, Gonabad University of Medical Sciences, Gonabad, Iran; ^5^Eye Research Center, The Five Senses Health Institute, Rassoul Akram Hospital, Iran University of Medical Sciences, Tehran, Iran; ^6^Faculty of Medicine, Jahrom University of Medical Science, Jahrom, Iran; ^7^Student Research Committee, School of Medicine, Tehran University of Medical Sciences, Tehran, Iran; ^8^Student Research Committee, Shiraz University of Medical Sciences, Shiraz, Iran; ^9^Student Research Committee, Virtual School of Medical Education and Management, Shahid Beheshti University of Medical Sciences, Tehran, Iran

**Keywords:** bullous pemphigoid, COVID-19 vaccines, COVID-19, flare-up, new-onset

## Abstract

**Background:**

Numerous cutaneous manifestations have been associated with the Coronavirus Disease 2019 (COVID-19) outbreak and vaccination, but new-onset bullous pemphigoid (BP) or flaring up of pre-existing BP is a rare side effect of COVID-19 vaccines that has been mentioned to a lesser extent in the literature. Therefore, we aimed to conduct a systematic review focused on the association between the new- onset or flare-up of BP and the COVID-19 vaccination.

**Method:**

A comprehensive literature search was conducted using PubMed (MEDLINE), Scopus, and the Web of Science databases up to 11 March 2023. The search aimed to identify English-language studies reporting new-onset or flare-ups of BP as a potential side effect of the COVID-19 vaccination. The search terms included bullous pemphigoid and COVID-19 vaccination-related MeSH terms.

**Results:**

The systematic review of 40 articles investigating the incidence of BP in individuals who received various COVID-19 vaccines revealed pertinent findings. Among the 54 patients with new-onset BP, the median age was 72.42 years, and most were men (64%). Conversely, the median age of the 17 patients experiencing a flare-up of BP was 73.35 years, with a higher proportion of women (53%). Regarding vaccination types, a significant number of patients (56%) developed new-onset BP after receiving the BNT162b2 vaccine (Pfizer-BioNTech).

**Conclusion:**

This study indicates a potential association between COVID-19 vaccinations, particularly mRNA vaccines, and the occurrence of BP. It suggests that this rare autoimmune disorder may be triggered as an adverse event following the COVID-19 vaccination. However, it is important to note that the majority of BP patients in our study were unaffected by the COVID-19 vaccine, and even those who experienced worsening of their conditions were managed without significant consequences. These findings provide additional evidence supporting the safety of COVID-19 vaccines. Physicians should be mindful of this uncommon adverse event and encourage patients to complete their planned vaccination schedules.

## 1 Introduction

Bullous pemphigoid (BP) is the most common type of blistering autoimmune disease associated with the elderly and is characterized by autoantibodies against BP180 and BP230 proteins at the basement membrane (BM) zone (1). Multiple factors, including radiation, ultraviolet (UV), trauma, burns, malignant disorders, and certain drugs, have been identified to trigger or exacerbate BP (2).

BP induced by vaccination against tetanus, diphtheria, poliomyelitis, whooping cough, influenza, meningococcal, pneumococcal, hepatitis B, rabies, and BCG has been reported in the literature (3–16), suggesting that vaccination might be related to the disease.

While the Food and Drug Administration (FDA) has licensed numerous coronavirus disease 2019 (COVID-19) medications, mass vaccination is still the most critical way to prevent the spread of the virus (17). Several COVID-19 vaccines are available, including nucleic acids, live-attenuated viruses, recombinant proteins, and inactivated viruses (17).

Numerous cutaneous manifestations have been associated with the COVID-19 outbreak and vaccination (18, 19), but new-onset BP or flaring up of the pre-existing BP is a rare side effect of COVID-19 vaccines that is mentioned to a lesser extent in the literature (20, 21).

To our knowledge, there is no systematic review or large-scale observational study specifically focused on the post-COVID-19 vaccination association with new-onset or flare-up BP. However, adverse cutaneous manifestations related to vaccines are rare, and most articles are case reports and case series. Therefore, we aimed to conduct a systematic review of all reported studies and consider the types of vaccine, the dose, and the days after vaccination when the disease occurs. Furthermore, the treatment of patients and the analysis of their pathological samples are clustered.

## 2 Methods

The current systematic review was conducted according to the Preferred Reporting Items for Systematic Reviews and Meta-Analyses (PRISMA) 2020 guidelines (22).

### 2.1 Literature search

The literature search was performed for English-language papers with no publication date restrictions in PubMed (MEDLINE), Scopus, and Web of Science databases (up to 23 March 2023) to identify publications reporting the BP new onset or flare-up following the COVID-19 vaccine treatment. To properly search the databases, the syntax was translated using Polyglot. Search terms included the keywords “COVID-19 vaccines OR SARS-CoV-2 Vaccines “, “BP,” and “case reports OR case series.” Boolean operators OR was used to combine synonyms of the keywords, and was used to combine search terms (the complete search formula is shown in the Supplementary section). To identify further eligible studies, we manually screened the first 30 pages using the Google Scholar search engine.

### 2.2 Study selection

Search results were exported to Endnote’s bibliographic management system (EndNote™ 20 for Windows, released on 30 October 2020), and duplicates were removed. The title and abstract of the papers were independently screened by two review authors (M.S. and N.K.), and irrelevant studies were excluded. After the initial screening, the remaining articles were reviewed in full text according to the inclusion and exclusion criteria. Discrepancies were reviewed by the corresponding author (M.A.E). We conducted all screening steps using EndNote 20 software.

The inclusion criteria for the current study were: (1) only case reports or case series studies; (2) reported cases of post-COVID-19 vaccination flare-up of pre-existing BP that were diagnosed according to international guidelines or biopsy findings; and (3) reported cases of post-COVID-19 vaccination new-onset BP manifestation that were diagnosed according to international guidelines or biopsy findings. The exclusion criteria were: (1) any other type of study except case reports and case series and (2) studies for which full texts were not available.

Overall, studies included in this systematic review were reports from cases or cases that developed new-onset BP or flare-up of the pre-existing BP after the COVID-19 vaccination. The diagnosis of the skin manifestation was BP. The outcome measurement was the association between the COVID-19 vaccine and BP.

### 2.3 Data extraction and risk of bias assessment

Two reviewers independently extracted data, and a third reviewer confirmed the consensus. The extracted data included information about the characteristics, objectives, and outcomes of each study. Lists were compared, and if there was controversy, a decision was made after discussion or with the help of a third reviewer. Two authors independently assessed the risk of bias among the included studies using the Joanna Briggs Institute (JBI) checklist, and a third reviewer confirmed the consensus. Case reports were evaluated using an 8-item JBI checklist that includes the patient’s demographic characteristics, description of diagnostic tests, medical history, treatment, current clinical condition, post-intervention clinical condition, adverse events, and the provision of takeaway lessons. For case series, the JBI checklist evaluates the 10-item inclusion criteria, condition measurement method, validity of diagnostic methods, whether participants were consecutively included, completeness of the inclusion process, reporting of demographic characteristics, clinical information, and outcomes, provision of clinic demographics, and evaluation of the appropriateness of the statistical analysis (23). Studies were classified into three quality classes: low, medium, or high risk of bias. The risk of bias was not used as an exclusion criterion in the selection of studies to provide a complete overview of the available data.

## 3 Results

### 3.1 Study selection

**Figure 1:**
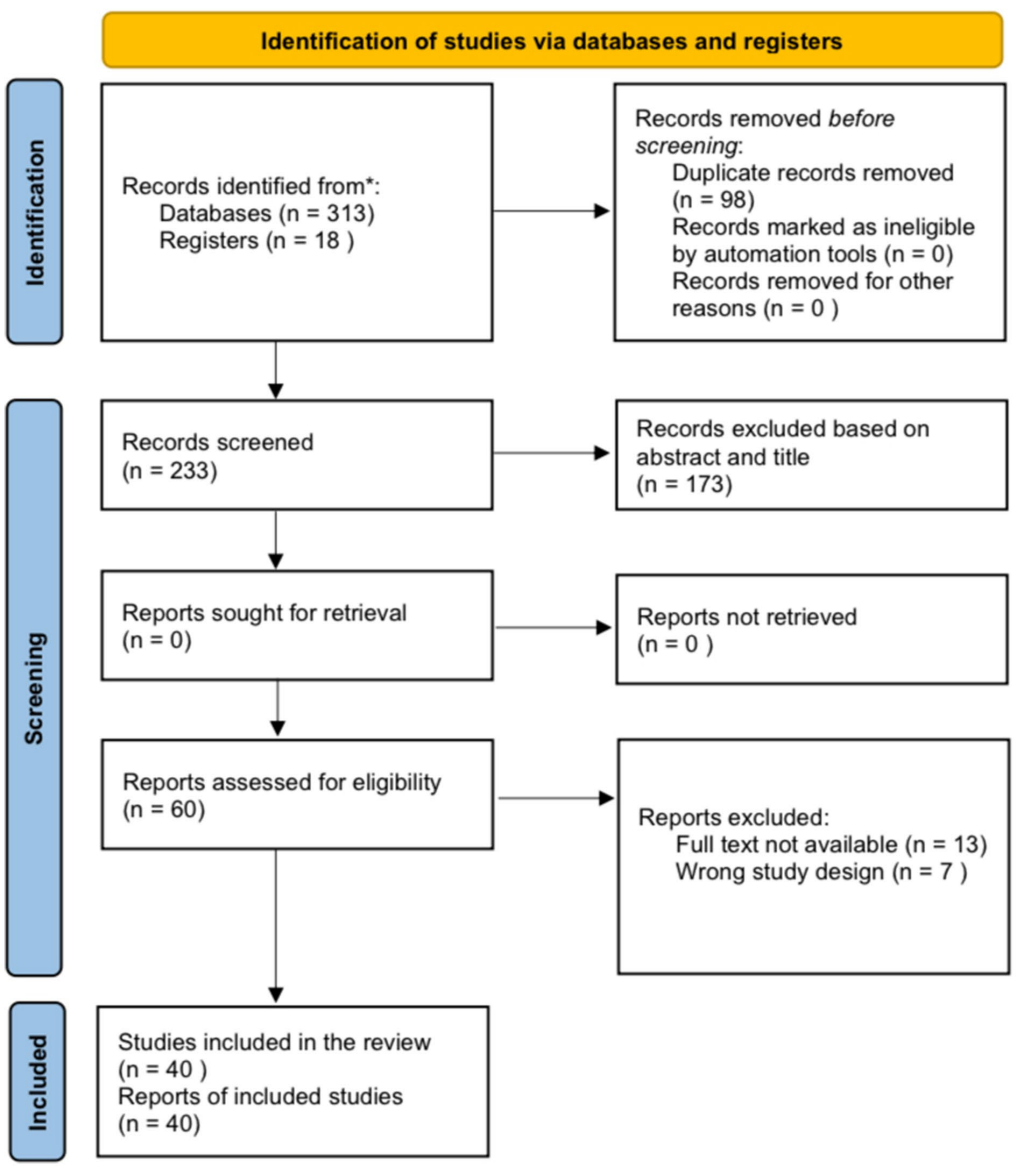
PRISMA flowchart.

All 313 records were identified through the systematic database search, and 18 records were identified through the manual search, of which 98 were detected as duplicates. In addition, after the title/abstract screening, 173 records were excluded. The full text of 13 articles was not found. Considering the eligibility criteria, seven studies were not case reports/case series and were therefore excluded. Finally, 40 reports met the inclusion criteria and were included in this review (Figure 1).

### 3.2 Study characteristics

Of the 40 included articles, 14 were case series and 26 were case reports, most of which were published in the form of letter-to-editor articles. Studies were published from 2021 to 2023 and peer-reviewed. Overall, 54 patients were diagnosed with BP, and 17 patients were identified as having BP flare (additional information about each patient is detailed in the Supplementary Files).

### 3.3 New-onset BP

The median age of the 54 new-onset BP patients was 72.42 years (ranging from 23 to 97 years old), with the majority being male (64%). BP lesions in patients are described as multiple itchy, tense bullae on an erythematous base with diffuse erythematous papules and urticarial plaques. These lesions are spread throughout the body, typically in the trunk area and extremities. The Nikolsky sign was also reported as positive in some studies. Finally, for the definitive diagnosis of BP, a biopsy was taken from the lesions, and in the investigations conducted by direct immunofluorescence (DIF), hematoxylin and eosin (H&E) staining, and enzyme-linked immunosorbent assay (ELISA) methods, the diagnosis of BP was confirmed.

#### 3.3.1 Clinical presentations

Psoriasis and dermatitis were among the most prevalent comorbidities, and more than half of the patients had no history of mucocutaneous disease (24–27). In addition, other mucocutaneous disorders such as leg ulcers, pemphigus vulgaris (PV), non-melanoma skin cancer, stasis dermatitis, and Raynaud syndrome were also seen in patients (20, 25, 28).

**Figure 2:**
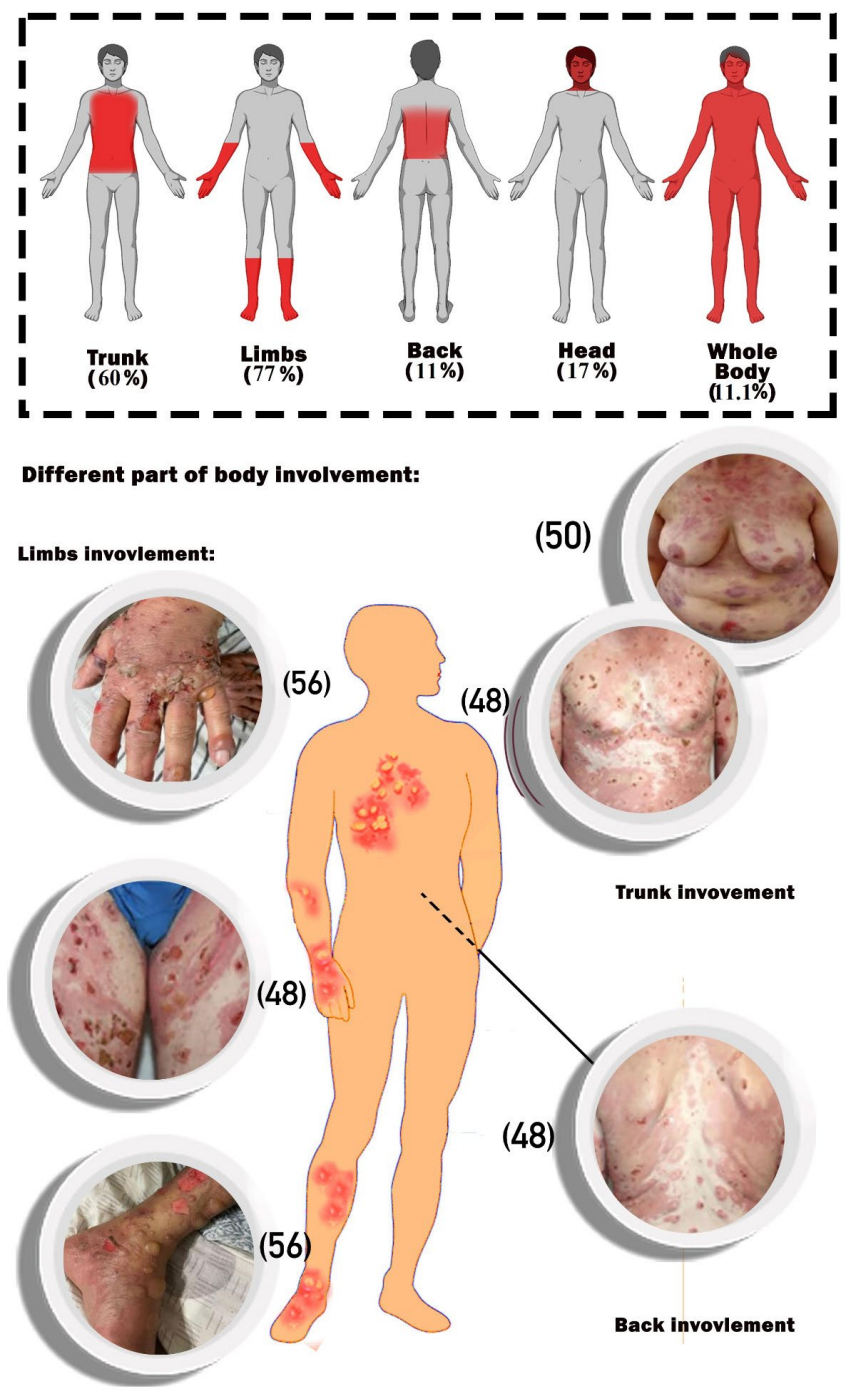
Schematic image of percentages of body parts involvement. The photos of the patients are extracted from the included articles and the reference number of the respective articles are (32–34). The photos of the patients are extracted from the included articles and the reference number of the respective articles are (48, 50, 56).

The main part of the complications in the body was the limbs, followed by the trunk. Whole-body involvement was seen in 11.1% of the patients. Furthermore, head involvement was seen in 17% of the cases (Figure 2).

As shown in Table 1, the majority of patients (56%) developed BP after receipt of the BNT162b2 vaccination, with the remainder developing it after receiving the mRNA1273 (11%), ChAdOx1-S [recombinant] (17%), and inactivated whole viral vaccines (17%). The majority of instances (51%) were documented following the first immunization dosage, and the rest after the second (35%) and third (14%) doses, respectively. Regarding the different types of COVID-19 vaccinations used in the study, among the mRNA vaccines were BNT1626b2 (Pfizer-BioNTech) and mRNA1273 (Moderna), among the recombinant vaccines was AstraZeneca, and among the dead-base viral vaccines were Sinopharm and CoronaVac, which have been injected into patients. Additionally, the median period between immunization and symptom onset was 15.24 days (range: 1–123 days). The most commonly reported BP lesions are in the upper and lower limbs (77%), trunk (60%), back (11%), head and neck (17%), and mucosal area (Figure 2).

**Table 1:** Demographic features and vaccination details of new-onset BP patients.

Variables			*N* ^*^
Patient characteristics	Median age	72.42 (23–97)	53
	Male/female	34 (64%)/19 (36%)	
Vaccine type	Pfizer-BioNTech (BNT162b2)	30 (56%)	54
	Moderna (mRNA-1273)	6 (11%)	
	AstraZeneca (ChAdOx1-S vaccine)	9 (17%)	
	Inactivated vaccines (CoronaVac, Sinopharm, and others)	9 (17%)	
Vaccine dose	First	25 (51%)	49
	Second	17 (35%)	
	Third	7 (14%)	
Distribution of lesions	extremities	27 (77%)	35
	Trunk	21 (60%)	
	Back	4 (11%)	
	Head and neck	6 (17%)	
	Other (genitalia, etc.)	4 (11%)	

*Number of reported patients.

Whole-body involvement was reported in six patients. One of them was an 89-year-old man who developed BP lesions all over his body 2 days after receiving his first dose of the Pfizer vaccine (29). After receiving the first dose of the AstraZeneca COVID-19 vaccine, a 71-year-old man experienced urticarial lesions on his arms that quickly spread to BP bullae throughout the rest of his body (30).

One death was noted: a 79-year-old man with whole-body BP lesions. Despite numerous attempts to treat his lesions with different therapies, the patient died of refractory septic shock secondary to pneumonia 6 months after exhibiting his first symptoms (4 months after receiving his first dose of rituximab) (31).

#### 3.3.2 Skin or mucosal biopsy and microscopic findings

H&E staining, ELISA, and DIF tests were carried out on the biopsy taken from the lesions to further confirm the diagnosis. Positive H&E staining was the most common histopathological finding, followed by significant eosinophilia and subepidermal blisters mixed with eosinophil infiltration in the DIF study.

#### 3.3.3 Management

**Figure 3:**
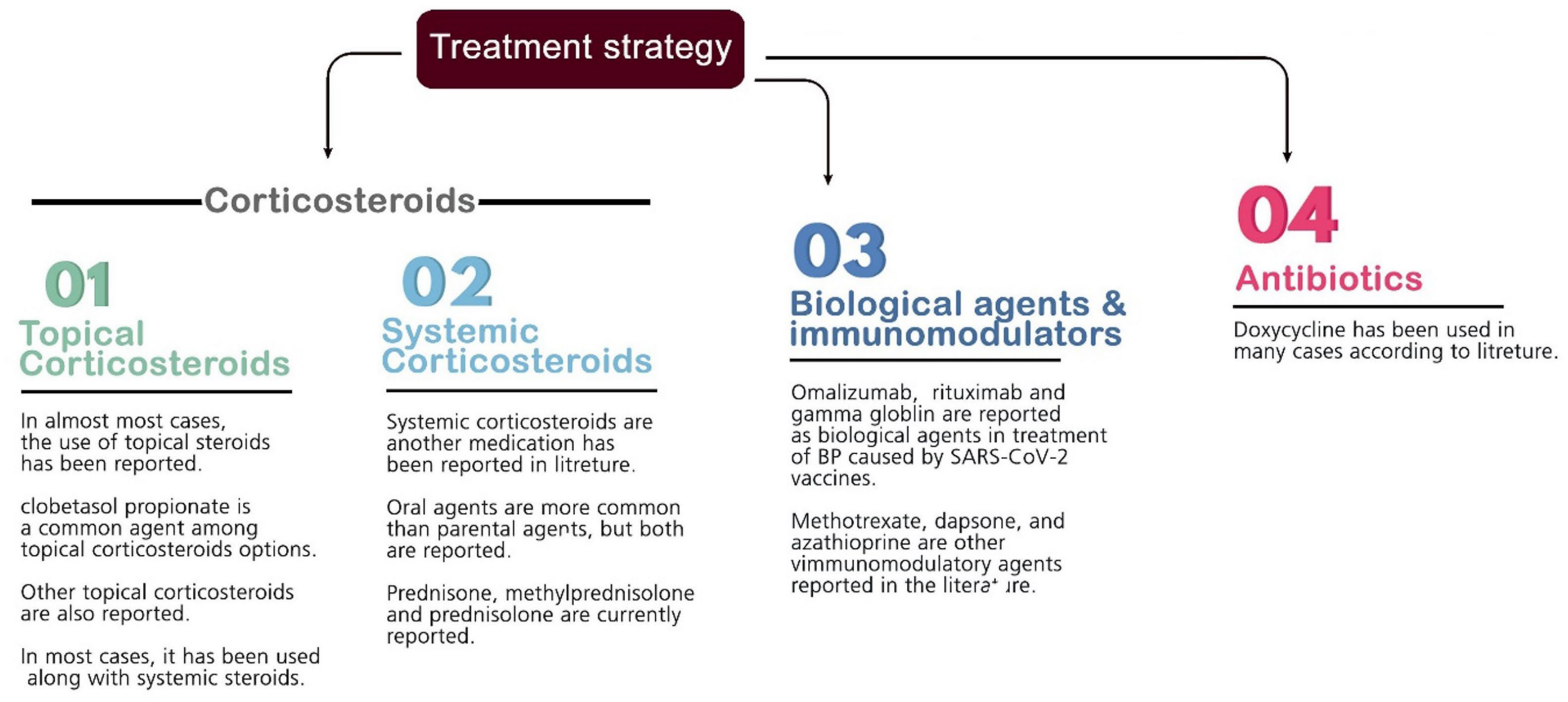
List of medications prescribed for disease management; the numbers are based on the most used drugs mentioned in the included articles.

Several medications are commonly used to suppress inflammation and treat BP lesions. Most patients were given prescriptions for systemic and topical corticosteroids as a pharmaceutical option to reduce inflammation (mainly prednisone, prednisolone, hydrocortisone, methylprednisolone, dapsone, dexamethasone, triamcinolone, and clobetasol). Other medications contain antibiotics (e.g., doxycycline), anti-parasites (permethrin), niacinamide, antihistamines (e.g., desloratadine and hydroxyzine), monoclonal antibodies (e.g., omalizumab and rituximab), immunosuppressive agents (azathioprine and methotrexate), intravenous immunoglobulin (IVIG), and different types of emollients. The most commonly used drug among the above treatments was different types of corticosteroids (systemic and topical) as monotherapy or in combination therapy (used for 91% of the patients). Furthermore, the average recovery time of the patients with the help of the above treatments is 45.15 days among the studies that reported this item (Figure 3).

### 3.4 BP flare

The median age of the 17 BP flare patients was 73.35 years (ranging from 57 to 86 years old), with the majority being female (53%). BP lesions in flare patients, also described as BP patients (several, pruritic small blistering lesions), were spread throughout the body, mostly across the limb and trunk. H&E staining, ELISA, and DIF tests were carried out on the biopsy taken from the lesions in the flare group, which helped to further confirm the diagnosis.

All patients were suffering from BP, which had been in the remission phase of the disease for a period of 6 months to 4 years before the injection of the vaccine. The BNT162b2 vaccine caused BP flare in the majority of patients (35%), while mRNA1273 (18%), ChAdOx1-S [recombinant] (12%), and inactivated whole viral vaccines (35%) caused it in the remaining patients. The majority of cases (8 patients, 57%) were reported after the first dose of the vaccination, with the remainder occurring after the second (4 patients, 29%) and third (2 patients, 14%) doses, respectively. Moreover, the median time between vaccination and flare-up onset was 22.35 days (range: 3–92 days). The trunk (71%) and limbs (57%) are the most commonly reported locations for the distribution of BP flare lesions. There were no reports of back, head, neck, and mucosal area involvement (The complete information about the patients is written in Supplementary Files).

### 3.5 Evidence map

**Figure 4:**
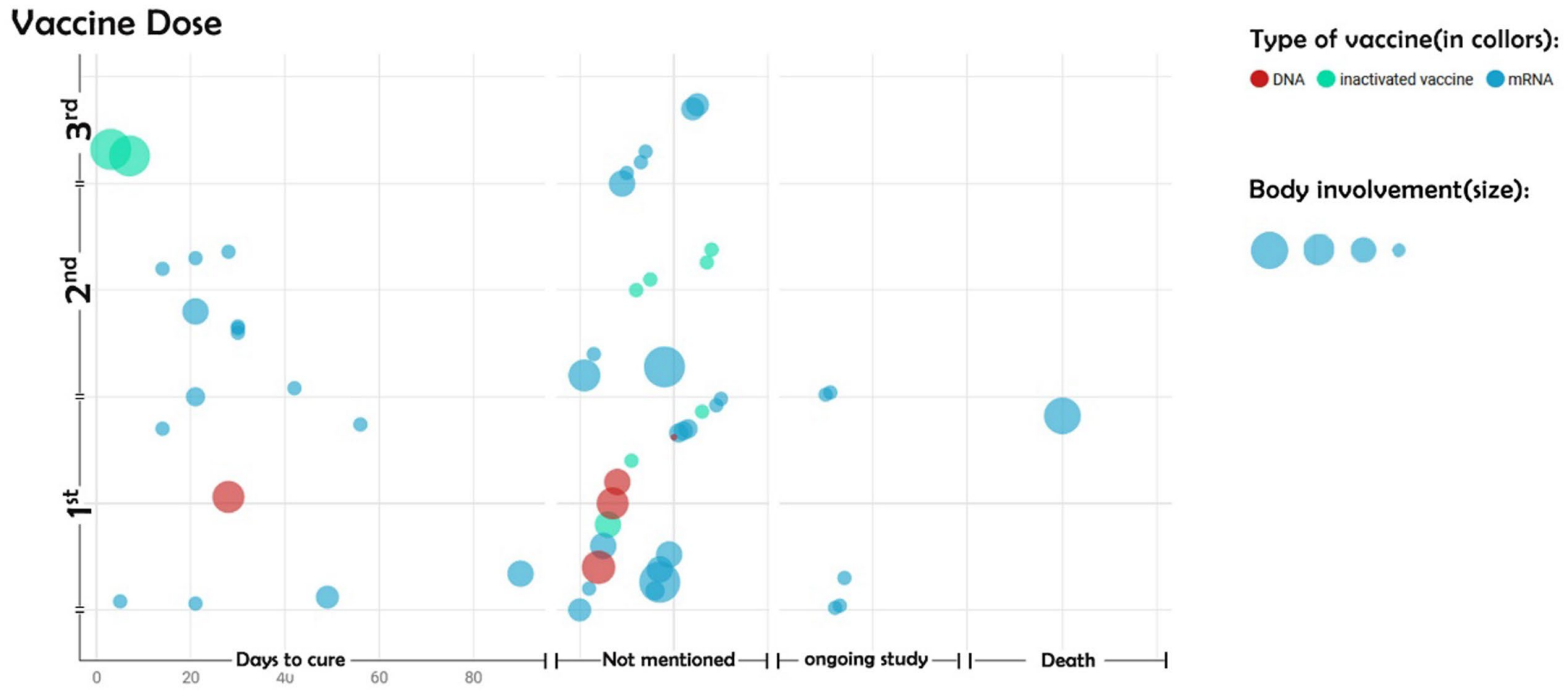
Size of body involvement based on the type of vaccine. The vertical axis represents the first, second, and third doses of the COVID-19 vaccine. The horizontal axis represents the days to cure the BP. Other parts of the horizontal axis indicate no mention of the duration of the disease cure, the continuation of the disease despite the completion of the study, or the death of the patients.

Complications of DNA vaccines mostly occurred following the first dose. In contrast, major dermal complications of inactivated vaccines developed after the third dose. mRNA vaccines were the only ones that caused large-sized dermal complications, followed by patient death (Figure 4). A noticeable number of studies did not report the time interval between vaccine injection and the appearance of dermal complications.

### 3.6 Quality assessment

Among the 26 included case reports, 2 reports scored perfect (8/8), and 1 report received the lowest quality score (4/8), with an overall mean score of 6.38. The most important scoring criteria included reporting the patients’ clinical condition on presentation and assessment methods or diagnostic tests, as well as the description of the adverse outcomes (harms) or unanticipated events. Studies reporting just the existence of takeaway lessons and intervention(s) or therapeutic procedure(s) received the lowest scores. Among 14 case series, 1 study received a 10/10 score, and 2 studies scored the lowest (5/10), with an overall mean score of 7.5.

## 4 Discussion

A comprehensive systematic review of case series and case report studies evaluated the occurrence of BP in individuals who received different available COVID-19 vaccines. The analysis revealed that the median age of BP patients was 72.42 years, with the majority being male (64%). However, in cases of BP flare-ups, a higher proportion of patients were female (53%). Regarding the types of vaccination, most patients (56%) developed BP after receiving the BNT162b2 vaccine (Pfizer-BioNTech). Furthermore, the BNT162b2 vaccine (Pfizer-BioNTech) was found to be the primary trigger for BP flare-ups in most patients (35%), while the mRNA1273 vaccine (18%) and ChAdOx1-S [recombinant] vaccine (12%) accounted for the remaining cases. Moreover, the median time intervals between immunization and the onset of symptoms were determined to be 15.24 days for new-onset BP cases and 22.35 days for BP flare-up patients.

BP lesions are predominantly distributed on the trunk (60%) and extremities (77%), although approximately 8.6% of patients experience involvement across their entire body. While most patients lacked a history of mucocutaneous disease, a prior dermatitis condition may be a potential predisposing factor for BP following vaccination.

The presence of autoantibodies against specific proteins of the hemidesmosome surface and subepidermal blistering characterizes BP, which is a member of the pemphigoid group (35). These circulating autoantibodies and tissue-bound components are BP hallmarks, which are against two specific autoantigens, namely collagen XVII (known as BP180) and dystonin-e (known as BP230) that exist as structural components on the hemidesmosomes and formations connecting the BM with the basal keratinocytes (36, 37).

The age factor evaluated in our study was 72.42 years, which represents the median age. This result matches the findings of the study by Marraza et al. (38). In addition, BP occurs mainly in the female population, which may be explained by the overexpression of the X-linked gene, which could mediate the stimulatory impact of estrogen and the antiviral response on the patient’s immune system (39). However, our study revealed different outcomes in sex demographic findings. Among our BP patients, it occurs mainly in men after vaccination, which is still unknown. However, the variety of predisposing conditions, such as past medical history, especially the history of specific kinds of dermatitis observed in nine patients in our included studies, type of used vaccines, and relevant genetic factors (40) could affect the final evaluation and might be the main reason for this difference (41).

The lesions observed in this study exhibited the typical and common clinical presentation associated with BP. This presentation is characterized by the appearance of inflammatory skin lesions that progress from erythematous patches to urticarial plaques, ultimately forming blisters on the trunk and extremities (42).

COVID-19 vaccines are available in a variety of forms, including nucleic acids, inactivated viruses, live-attenuated viruses, and recombinant proteins, which have been approved for usage or are still in clinical trials (17, 43). The mRNA vaccines are the first COVID-19 vaccines licensed in the United States. Two mRNA vaccines, BNT162b2 and mRNA1273, encode spike protein that is encapsulated in lipid nanoparticles, which promotes receptor recognition and viral entry into human cells (17, 29, 44). As more people receive vaccines against COVID-19, the reported delayed cutaneous reactions are likely to increase. A recent study described various delayed cutaneous reactions to COVID-19 vaccines or flares of pre-existing dermatologic conditions such as eczema, lichen planus, atopic dermatitis, psoriasis, and urticarial vasculitis (45–47). Flare-up of pre-existing BP or manifestation of new-onset BP is a rare side effect of COVID-19 vaccines and has also been reported following rabies, influenza, and hepatitis B vaccinations (5, 8, 15). In this study, we found that BP is more reported after mRNA COVID-19 vaccines than other types of vaccines, particularly among the elderly.

Moreover, individuals who received the BNT162b2 vaccine, especially the first dose, were more susceptible to BP than those receiving the other types of vaccines.

An article by Shakoei et al. (48) showed that after receiving the first dose of Sinopharm vaccine, BP and PV (pemphigus vulgaris) was seen in vaccinated people. This phenomenon’s underlying pathogenesis could be explained by molecular mimicry between the spike proteins of SARS-CoV-2 and specific proteins present in the human BM, such as BP180 and BP230. This molecular mimicry may produce autoantibodies, resulting in the development or exacerbation of autoimmune diseases such as BP (29, 49, 50). However, other scientists hypothesized that the vaccine might cause a strong immune response in people with immunological predispositions due to the activation of T-helper cells and the generation of antibodies (32).

T-helper lymphocytes have an essential role in the immune system’s function by producing several immune factors and cytokines, which activate other parts of the human immune system, especially by inducing B-cell lymphocytes to produce antibodies. CD4 is an important protein expressed on the surface of T-helper cells, playing a critical role in recognizing unfamiliar substances. In addition, the mRNA vaccines have stimulated the human immune system by producing antibodies from B-cell immune and activated T-helper lymphocyte cells, which might cause autoimmune drug-induced BP events (51). Moreover, B-cells produced autoantibodies when faced with the BP180 antigen of the coronavirus vaccine, and a B-cell immune response happened. Then, the skin BM cell layer was destroyed based on protease, which was released from the immune complement pathway. The mechanism illustrated that humeral immunity has a significant role in COVID-19 vaccine-induced BP (33).

Furthermore, it suggested that COVID-19 vaccines induced the production of some crucial cytokines in autoimmune diseases, such as IL-4, IL-17, and IL-21, which are linked to germinal center activation and play a critical role in the early phases of autoimmune diseases (52, 53). IL-4 can stimulate the production of IgG, IgE, and class switch recombination (CSR) (54). IL-17 and IL-21 promote B-cell proliferation and increase the production of antibodies, and IL-17 also causes inflammatory responses by increasing the migration of myeloid cells into the tissue (34, 57–60).

Most individuals who present medication-induced BP have received a type of mRNA vaccine, of which Pfizer-BioNTech has the highest number. The reason for that may be possible due to two hypotheses. First of all, the mechanism of the vaccine is not well understood and triggers the immune system, which induces autoimmune BP. On the other hand, a high number of people have received the first safe vaccine, which the FDA has authorized; in other words, most of the participants were injected with the Pfizer-BioNTech COVID-19 vaccine (56–60).

In some of the included articles, BP occurred less than 1 week after the COVID-19 vaccine injection. Meanwhile, antibody production needs approximately 1 week. Due to the fact that all the case reports and case series studies that were written in this field and received enough points of risk of bias assessment were included in the current study, their data were written. However, it has not been clearly proven whether BP can happen less than a week after the injection of the COVID-19 vaccine. For this reason, the occurrence of BP 1 week after the injection of the vaccine is uncertain.

Moreover, skin biopsies of patients who experienced COVID-19 vaccine-induced BP revealed the presence of IgG, IgM, and C3 deposits along the hemidesmosomes and epithelial anchors of the BM. Additionally, the biopsies showed scattered eosinophils and perivascular infiltrates (42, 56).

The management of BP post-vaccination was similar to other medications commonly used to reduce inflammation and control BP lesions. We noted that many patients with BP were treated with systemic and topical corticosteroids as monotherapy or combination therapy. Some other medications, such as doxycycline, methotrexate, and azathioprine, were also used as add-on therapy. A study by Shakoei et al. (45) showed that remission was observed with 4 mg dexamethasone three times daily in a pemphigus flare-up. Avallone et al. (28) reported PV present BP 5 days after the BNT162b2 vaccine and resolution after 10 months with oral prednisone and azathioprine.

It is worth mentioning that the COVID-19 vaccine did not significantly impact the majority of BP patients. Most BP patients who received the COVID-19 vaccine did not experience an exacerbation, and only a few had an exacerbation. Even those who experienced an exacerbation were successfully managed without significant consequences. On the other hand, out of the 13 billion doses of COVID-19 vaccines administered worldwide, approximately 5.33 billion individuals have received at least one dose, corresponding to approximately 69.5% of the global population (59, 60). These few people who experienced new-onset BP are small compared to the large number of people who were vaccinated, and these findings provide additional reassurance regarding the safety of COVID-19 vaccines.

## 5 Conclusion

This review suggests a potential association between COVID-19 vaccines, particularly mRNA vaccines, and the occurrence of BP, a rare autoimmune disease. However, the exact correlation between the development of bullous reactions and the vaccine administration remains unclear, necessitating further investigation. It is important to emphasize that in our study, the COVID-19 vaccine did not significantly impact the majority of BP patients, and even those who experienced exacerbations were successfully managed without significant consequences. On the other hand, these few people who experienced new-onset BP are small compared to the large number of people who were vaccinated. These findings provide additional reassurance regarding the safety of COVID-19 vaccines. Physicians must remain vigilant regarding this uncommon adverse event, particularly regarding vaccination campaigns and exposure to infectious agents, while encouraging patients to complete their recommended vaccine schedules.

## Data availability statement

The original contributions presented in the study are included in the article/Supplementary material, further inquiries can be directed to the corresponding author.

## Author contributions

AG: Data curation, Writing – original draft. MS: Data curation, Writing – original draft. HH: Writing – original draft. AM-S: Data curation, Writing – original draft. AA: Methodology, Writing – review & editing. KK: Data curation, Writing – original draft. MB: Software, Writing – original draft. AK-J: Writing – review & editing. MD: Methodology, Writing – review & editing. NK: Data curation, Writing – original draft. AB: Methodology, Writing – original draft. ME: Conceptualization, Methodology, Project administration, Supervision, Writing – original draft, Writing – review & editing.
