# Supplementary material for: New-onset or flare-up of bullous pemphigoid associated with COVID-19 vaccines: a systematic review of case report and case series studies
Source: Front Med (Lausanne). 2024 Apr 8;11:1293920. doi: 10.3389/fmed.2024.1293920 (PMC11036870; doi:10.3389/fmed.2024.1293920)
Supplement: Supplementary file 1 [file Table_1.DOCX]

**Search strategy**

**Date: 23 March 2023**

| **Database** | **Strategy** | **Number** |
| --- | --- | --- |
| PubMed | (“Bullous Pemphigoid”[ALL Fields] OR pemphigoid[ALL Fields] OR (Pemphigoid[ALL Fields] AND Bullous[ALL Fields)) AND ("COVID-19 Vaccines"[ ALL Fields] OR "COVID 19 Vaccines"[ ALL Fields] OR "COVID-19 Virus Vaccines"[ ALL Fields] OR "COVID 19 Virus Vaccines"[ ALL Fields] OR "COVID-19 Virus Vaccine"[ ALL Fields] OR "COVID 19 Virus Vaccine"[ ALL Fields] OR "COVID19 Virus Vaccines"[ ALL Fields] OR "COVID19 Virus Vaccine"[ ALL Fields] OR "COVID19 Vaccines"[ ALL Fields] OR "COVID19 Vaccine"[tiab] OR "SARS-CoV-2 Vaccines"[tiab] OR "SARS CoV 2 Vaccines"[tiab] OR "SARS-CoV-2 Vaccine"[ ALL Fields] OR "SARS CoV 2 Vaccine"[ ALL Fields] OR "SARS2 Vaccines"[ ALL Fields] OR "SARS2 Vaccine"[ ALL Fields] OR "Coronavirus Disease 2019 Vaccines"[ ALL Fields] OR "Coronavirus Disease 2019 Vaccine"[tiab] OR "Coronavirus Disease 2019 Virus Vaccine"[tiab] OR "Coronavirus Disease 2019 Virus Vaccines"[tiab] OR "Coronavirus Disease-19 Vaccines"[tiab] OR "Coronavirus Disease 19 Vaccines"[tiab] OR "Coronavirus Disease-19 Vaccine"[tiab] OR "Coronavirus Disease 19 Vaccine"[tiab] OR "COVID 19 Vaccine"[tiab] OR "2019-nCoV Vaccine"[tiab] OR "2019 nCoV Vaccine"[tiab] OR "2019 Novel Coronavirus Vaccines"[tiab] OR "2019 Novel Coronavirus Vaccine"[tiab] OR "2019-nCoV Vaccines"[tiab] OR "2019 nCoV Vaccines"[tiab] OR "COVID-19 Vaccine"[tiab] OR "SARS Coronavirus 2 Vaccines"[tiab] OR "2019 nCoV Vaccine mRNA 1273"[tiab] OR "Moderna COVID-19 Vaccine"[tiab] OR "Moderna COVID 19 Vaccine"[tiab] OR "Elasomeran"[tiab] OR "Moderna COVID-19 Vaccine RNA"[tiab] OR "Moderna COVID 19 Vaccine RNA"[tiab] OR "COVID-19 Vaccine Moderna"[tiab] OR "COVID 19 Vaccine Moderna"[tiab] OR mRNA-1273[tiab] OR "mRNA 1273"[tiab] OR TAK-919[tiab] OR "TAK 919"[tiab] OR TAK919[tiab] OR M-1273[tiab] OR "M 1273"[tiab] OR M1273[tiab] OR mRNA-1273.211[tiab] OR "mRNA 1273.211"[tiab]) | 57 |
| Scopus | (“Bullous Pemphigoid” OR “pemphigoid” OR (“Pemphigoid” AND “Bullous”)) AND ("COVID-19 Vaccines" OR "COVID 19 Vaccines" OR "COVID-19 Virus Vaccines" OR "COVID 19 Virus Vaccines" OR "COVID-19 Virus Vaccine" OR "COVID 19 Virus Vaccine" OR "COVID19 Virus Vaccines" OR "COVID19 Virus Vaccine" OR "COVID19 Vaccines" OR "COVID19 Vaccine" OR "SARS-CoV-2 Vaccines" OR "SARS CoV 2 Vaccines" OR "SARS-CoV-2 Vaccine" OR "SARS CoV 2 Vaccine" OR "SARS2 Vaccines" OR "SARS2 Vaccine" OR TITLE-ABS-KEY("Coronavirus Disease 2019 Vaccines") OR TITLE-ABS-KEY("Coronavirus Disease 2019 Vaccine") OR TITLE-ABS-KEY("Coronavirus Disease 2019 Virus Vaccine") OR TITLE-ABS-KEY("Coronavirus Disease 2019 Virus Vaccines") OR TITLE-ABS-KEY("Coronavirus Disease-19 Vaccines") OR TITLE-ABS-KEY("Coronavirus Disease 19 Vaccines") OR TITLE-ABS-KEY("Coronavirus Disease-19 Vaccine") OR TITLE-ABS-KEY("Coronavirus Disease 19 Vaccine") OR TITLE-ABS-KEY("COVID 19 Vaccine") OR TITLE-ABS-KEY("2019-nCoV Vaccine") OR TITLE-ABS-KEY("2019 nCoV Vaccine") OR TITLE-ABS-KEY("2019 Novel Coronavirus Vaccines") OR TITLE-ABS-KEY("2019 Novel Coronavirus Vaccine") OR TITLE-ABS-KEY("2019-nCoV Vaccines") OR TITLE-ABS-KEY("2019 nCoV Vaccines") OR TITLE-ABS-KEY("COVID-19 Vaccine") OR TITLE-ABS-KEY("SARS Coronavirus 2 Vaccines") OR TITLE-ABS-KEY("2019 nCoV Vaccine mRNA 1273") OR TITLE-ABS-KEY("Moderna COVID-19 Vaccine") OR TITLE-ABS-KEY("Moderna COVID 19 Vaccine") OR TITLE-ABS-KEY("Elasomeran") OR TITLE-ABS-KEY("Moderna COVID-19 Vaccine RNA") OR TITLE-ABS-KEY("Moderna COVID 19 Vaccine RNA") OR TITLE-ABS-KEY("COVID-19 Vaccine Moderna") OR TITLE-ABS-KEY("COVID 19 Vaccine Moderna") OR TITLE-ABS-KEY(“mRNA-1273”) OR TITLE-ABS-KEY("mRNA 1273") OR TITLE-ABS-KEY(“TAK-919”) OR TITLE-ABS-KEY("TAK 919") OR TITLE-ABS-KEY(“TAK919”) OR TITLE-ABS-KEY(“M-1273”) OR TITLE-ABS-KEY("M 1273") OR TITLE-ABS-KEY(“M1273”) OR TITLE-ABS-KEY(“mRNA-1273.211”) OR TITLE-ABS-KEY("mRNA 1273.211")) | 221 |
| Web of Science | ((ALL=(“Bullous Pemphigoid”)) OR (ALL=(“Pemphigoid”))) AND (((((((((((((((((((((((((((((((((((((((((((((((((((ALL=("COVID-19 Vaccines")) OR ALL=("COVID 19 Vaccines")) OR ALL=("COVID-19 Virus Vaccines")) OR ALL=("COVID 19 Virus Vaccines")) OR ALL=("COVID-19 Virus Vaccine")) OR ALL=("COVID 19 Virus Vaccine")) OR ALL=("COVID19 Virus Vaccines")) OR ALL=("COVID19 Virus Vaccine")) OR ALL=("COVID19 Vaccines")) OR ALL=("COVID19 Vaccine")) OR ALL=("SARS-CoV-2 Vaccines")) OR ALL=("SARS CoV 2 Vaccines")) OR ALL=("SARS-CoV-2 Vaccine")) OR ALL=("SARS CoV 2 Vaccine")) OR ALL=("SARS2 Vaccines")) OR ALL=("SARS2 Vaccine")) OR TS=("Coronavirus Disease 2019 Vaccines")) OR TS=("Coronavirus Disease 2019 Vaccine")) OR TS=("Coronavirus Disease 2019 Virus Vaccine")) OR TS=("Coronavirus Disease 2019 Virus Vaccines")) OR TS=("Coronavirus Disease-19 Vaccines")) OR TS=("Coronavirus Disease 19 Vaccines")) OR TS=("Coronavirus Disease-19 Vaccine")) OR TS=("Coronavirus Disease 19 Vaccine")) OR TS=("COVID 19 Vaccine")) OR TS=("2019-nCoV Vaccine")) OR TS=("2019 nCoV Vaccine")) OR TS=("2019 Novel Coronavirus Vaccines")) OR TS=("2019 Novel Coronavirus Vaccine")) OR TS=("2019-nCoV Vaccines")) OR TS=("2019 nCoV Vaccines")) OR TS=("COVID-19 Vaccine")) OR TS=("SARS Coronavirus 2 Vaccines")) OR TS=("2019 nCoV Vaccine mRNA 1273")) OR TS=("Moderna COVID-19 Vaccine")) OR TS=("Moderna COVID 19 Vaccine")) OR TS=("Elasomeran")) OR TS=("Moderna COVID-19 Vaccine RNA")) OR TS=("Moderna COVID 19 Vaccine RNA")) OR TS=("COVID-19 Vaccine Moderna")) OR TS=("COVID 19 Vaccine Moderna")) OR TS=(mRNA-1273)) OR TS=("mRNA 1273")) OR TS=(TAK-919)) OR TS=("TAK 919")) OR TS=(TAK919)) OR TS=(M-1273)) OR TS=("M 1273")) OR TS=(M1273)) OR TS=(mRNA-1273.211)) OR TS=("mRNA 1273.211")) | 35 |
