# Supplementary material for: New-onset or flare-up of bullous pemphigoid associated with COVID-19 vaccines: a systematic review of case report and case series studies
Source: Front Med (Lausanne). 2024 Apr 8;11:1293920. doi: 10.3389/fmed.2024.1293920 (PMC11036870; doi:10.3389/fmed.2024.1293920)
Supplement: Supplementary file 2 [file Table_2.docx]

| Authors (year) | Case age | Case gender | Patients' mucocutaneous disease history | Patients' other comorbidity | Drug history at the time of vaccination | Vaccine type | Vaccine dose | Description of mucocutaneous reactions | Distribution | reactions onset | Skin or mucosal biopsy | Diagnosis | Resolution after (time) | Management of reactions | Ref |
| --- | --- | --- | --- | --- | --- | --- | --- | --- | --- | --- | --- | --- | --- | --- | --- |
| Fouad Alshammari et al. (2022) | 78 | M | NM | diabetes, hypertension, and chronic renal disease | NM | Pfizer Covid-19 vaccine | second dosage | numerous bullae  and  pruritus and urticated plaques | extremities | one day | IgG (+1), IgM (+1), and C3 (+1) staining of the basement membrane  modest perivascular lymphocytic infiltrative and scattered eosinophils | drug-induced bullous pemphigoid  and severe sepsis due to a skin infection  pulmonary embolism | 3 weeks  death one month after discharge from our hospital | topical and systemic corticosteroids, fusidic acid cream, and emollients  meropenem and vancomycin  heparin infusion | (1) |
| Muhamad Khalid et al. (2021) | 62 | M | NM | seasonal allergies, hypercholesteremia, and mental retardation | risperidone atorvastati  loratadine | Moderna COVID-19 vaccine | first dose | worsening of the blisters and redness to the with larger blisters | anterior chest, genitalia, bilateral hands, and bilateral lower feet | 2 weeks | erythema multiforme, bullous pemphigoid and Stevens-Johnson Syndrome  presence of a significant number of eosinophils | Bullous Pemphigoid | NM | Topical corticosteroid  Systemic corticosteroid  Steroid-sparring immunosuppressant  Immunomodulator | (2) |
| Israel Pérez-López et al. (2021) | 78 | F | NM | diabetes mellitus and Alzheimer's disease | Memantine  insulin | Comirnaty and Pfizer-BioNTech COVID-19 Vaccine | second dose | tense blisters on an erythematous base | facial area, trunk, and extremities | three days | skin biopsy was diagnostic of bullous pemphigoid  slight eosinophilia | bullous pemphigoid | NM | prednisone 40 mg every 24 | (3) |
| Valerie Larson et al. (2022) | 76 | M | NM | NM | NM | Pfizer‐BioNTech | first dose | Clustered and tense bullae | NM | 3 wk | Subepidermal bulla and a superficial interstitial infiltrate with eosinophils | Bullous pemphigoid | NM | prednisone, topical corticosteroids, doxycycline, niacinamide, and antihistamines | (4) |
| Valerie Larson et al. (2022) | 84 | M | NM | NM | NM | Moderna | second dose | Widespread erythematous papules and plaques, some with vesiculation | NM | 2 wk | Intraepidermal spongiotic vesicles with eosinophilic spongiosis | Bullous pemphigoid | NM | oral prednisone and topical corticosteroids | (4) |
| Mary M Tomayko et al. (2021) | 97 | F | Psoriasis |  |  | Pfizer | Dose 2 |  |  | d 2 | IgG/C3/IgA  SSS, roof | Bullous pemphigoid | wk 2 | TCS, DCN, NAM |  |
| F-Z Agharbi et al. (2022) | 77 | NM | NM | NM | NM | AstraZenca | First dose | diffuse itchy bullous eruption | Trunk, scalp, limbs | 24 h | IgG | Bullous pemphigoid | NM | high potency topical  steroids (propionate of clobetasol 0.05% cream) once a day and  doxycycline (100 mg/day) | (5) |
| W-K Hung and C-C Chi (2022) | 39 | M | Psoriasis | NM | topicalChinese medicine (indigo naturalis) | Moderna | First dose | multiplepruritic and tense bullae on bilateral hands and feet, red urticarialpapules and plaques on the trunk | Limbs, trunk | 1 month | subepidermal bulla containing eosinophilicinfiltration and focal re-epithelialization.  C3, IgG.  Indirect immunofluor-escence analysis: positive titre of 1 : 40 for anti-basement membrane zone antibodies | Bullous pemphigoid | NM | Oral methylpred-nisolone  Intravenousmethylprednisolon  ral methylprednisolone  doxycycline | (6) |
| Ecem Bostan et al. (2021) | 67 | M | NM | diabetes mellitus and benign prostate hyperplasia | vildagliptin plus metformin, tam-sulosin | Inactivated Covid-19 | First dose | widespread diffuse erythematous rash accompanied by bullae and intensive pruritus,  superficial erosion and hemorrhagic/seropurulent crust formation on the trunk, upper extremities andthighs upon erythematous, confluent patches and plaques | trunk, upper extremities andthighs | 5 weeks | C3, IgG | Bullous pemphigoid | NM | Prednisolone,  omalizumab | (7) |
| Carlo Alberto Maronese et al. (2022) | 85 | M | NM | NM | sitagliptin | Pfizer | Second dose | the rash consisted of tense bullae lacking the characteristic erythematous base of classic BP and excoriated lesions | NM | 3-4 weeks since 1^st^ dose until BP onset | C3, IgG.  ELISA: BP180(44 U/ml)  BP230(21 U/ml) | Bullous pemphigoid | 1 month | DPP4-i discontinuation, topical clobetasol and either oral doxycycline | (8) |
| Carlo Alberto Maronese et al. (2022) | 84 | F | NM | NM | Linagliptin | Pfizer | First dose | NM | NM | 4 weeks since 1^st^ dose until BP onset | ELISA: negative results  BP180(11.5 U/ml)  BP230(3.6 U/ml) | Bullous pemphigoid | 1 month | prednisone | (8) |
| Carlo Alberto Maronese et al. (2022) | 86 | M | NM | NM | Linagliptin | Pfizer | First dose | NM | NM | 2 weeks since 1^st^ dose until BP onset | ELISA:  BP180(20.9 U/ml)  BP230(0.4 U/ml) | Bullous pemphigoid | 1 month | prednisone | (8) |
| Massimo Dell'Antonia et al. (2021) | 83 | M | NM | hypertension | Perindopril, amlodipine | Pfizer | First dose | several erosions and tense bullae on erythematous base | limbs, trunks | 1 week | C3 | Bullous pemphigoid | 3 weeks | prednisone | (9) |
| Fouzia Hali Sr. et al. (2022) | 51 | M | NM | NM | NM | Oxford AstraZenca | First dose | diffuse tense bullae associated with urticarial plaques, post-bullous erosions and cocarde-like lesions, multiple erosions on the buccal mucosa. Nikolsky sign was positive | trunk, back, and legs | 1 week | C3, IgG, PB180. | Bullous pemphigoid | 4 weeks | prednisone | (10) |
| Fouzia Hali Sr. et al. (2022) | 54 | F | NM | NM | NM | Oxford AstraZeneca | First dose | Rash, localized blisters, tense bullae associated with urticarial plaques with a positive Nikolsky sign. | both arms, abdomen, back, thighs, lower legs | 3 days | C3, IgG | Bullous pemphigoid | NM | clobetasol propionate 0.05% | (10) |
| Fouzia Hali Sr. et al. (2022) | 68 | M | NM | hypercholesterolemia | NM | Oxford AstraZeneca | First dose | tense bullae and post-bullous erosions on an erythematous base, with a positive Nikolsky sign | limbs and trunk | 2 weeks | C3 | Bullous pemphigoid | NM | oral corticosteroid | (10) |
| Federico Bardazzi et al. (2022) | 76 | F | leg ulcers | NM | NM | Pfizer | Third dose | pruritic bullous eruption | Right leg, back | 12 days | ELISA: BP180 (48 U/ml)  BP320 (24 U/ml) | Bullous pemphigoid | NM | Methylprednisolone initial dose of 0.5 mg/kg/die for 5 days and then gradually tapered + topical high potency steroids twice daily | (11) |
| Federico Bardazzi et al. (2022) | 79 | M | NM | NM | NM | Pfizer | Third dose | NM | NM | 9 days | ELISA: BP180 (200 U/ml)  BP320 (19 U/ml) | Bullous pemphigoid | NM | Methylprednisolone initial dose of 0.5 mg/kg/die for 5 days and then gradually tapered + oral nicotinamide 750 mg/daily + topical high potency steroids once daily | (11) |
| Safoura Shakoei et al. (2022) | 85 | F | NM | NM | NM | Sinopharm | first dose | Generalized  cutaneous blisters  and ulcers, pruritus | NM | 20 days | NM | Bullous pemphigoid | NM | Topical clobetasol and  oral doxycycline | (12) |
| Safoura Shakoei et al. (2022) | 91 | M | NM | NM | NM | Sinopharm | first dose | Mucocutaneous  ulcers | Pruritus | 19 days | NM | Bullous pemphigoid | NM | Topical Clobetasol,  Rituximab | (12) |
| G. Avallone et al. (2022) | 46 | M | pemphigus vulgaris | NM | NM | BNT162b2 vaccine | First dose | Small erosive lesions | oral mucosa | 5 days | anti-desmoglein antibodies | Bullous pemphigoid | 10 months | oral prednisone and azathioprine | (13) |
| Pauluzzi M. et al. (2022) | 46 | M | NM | NM | NM | COVID-19 mRNA BNT162b2 vaccine (Comirnaty/Pfizer) | First dose | wide-spread itchy erythema along with sev-eral tense blisters | Trunk, Arms | 15 days | ubepidermal detachmentalong with an eosinophilic inflammatory cell infiltrate in thesuperficial dermis  C3, BP180 | Bullous pemphigoid | 7 weeks | intra-muscular methylprednisolone acetate, oral azathioprine oral azathioprine, oral methylprednisolone | (14) |
| Gambichler T. et al. (2022) | 80 | M | NM | NM | NM | BTN162b2 | First dose | reddish itchy macules with small blister, erythematous/bullous lesion | Lower leg, trunk | 1 week | Subepidermal cleft.  IgG, C3  BP180:365  BP320:223  high clonality of Tcells | Bullous pemphigoid | NM | NM | (15) |
| Gambichler T. et al. (2022) | 89 | M | NM | NM | NM | BTN162b2 | First dose | itchy erythematous/bullous lesions | Entire integument | 2 days | Subepidermal cleft.  IgG, C3  BP180:115  BP320:41  high clonality of Tcells | Bullous pemphigoid | NM | NM | (15) |
| Roberto Russo et al. (2022) | 75 | M | NM | type-II diabetes mellitus | gliptins | Comirnaty Pfizer-BioNTech | First dose | tense blisters on erythematous skin | NM | 48 h | NM | Bullous pemphigoid | Prompt clinical improvement | Discontinuation of gliptins, the cutaneous lesions were managed by topical steroids | (16) |
| Young J. et al. (2022) | 68 | M | NM | NM | NM | Pfizer | First dose | Blisters. intense, generalized pruritus | Sternal area, right side of the chest, upper back | 3 days | eosinophils and hemosiderophages.  IgG and C3. | Bullous pemphigoid | 3 months | acyclovir for a pre-sumed diagnosis of herpes zoster with no improvement and laterdesloratadine and a oral prednisolone | (17) |
| Saerrah Murryam | 68 | F | NM | A background of chronic obstructive pulmonary disease and  hypercholesterolaemia | NM | Pfizer | First dose | tense bullae, severe pruritus | a localized distribution  confined to the dorsal aspect of her hands, forearms and ears  only | 5 days | IgG and C3  anti-BP180 (+)  anti-BP230 (+) | Bullous pemphigoid | NM | oral prednisolone and doxycycline | (18) |
| Kenta Nakamura et al. (2021) | 83 | F | xerotic eczema | Bipolar disorder | NM | tozinameran, the BNT162b2 mRNA COVID‐19 vaccine | Second dose | erythema and blisters | All over the body | 3 days | subepidermal blisters with eosinophil infiltration.  IgG | Bullous pemphigoid | NM | oral prednisolone (didn’t improve the condition)  steroid pulse therapy and high‐dose i.v. immunoglobulin therapy | (19) |
| Yingjie Zhang et al. (2022) | 23 | M | eczema | NM | NM | inactivated COVID‐19 vaccine (Changchun Institute of Biological Products Co., Ltd., Changchun, China) | Third dose | pruritic and tense bullae | First: **bilateral upper limbs**  Then spreading over the **entire body** within 10 days | 1 day | subepidermal blister formation and an inflammatory infiltrate mainly composed of eosinophils in the dermis and bulla cavity  IgG and C3.  positive titer of 1:10 for antibasement membrane zone antibodies.  BP180: 1:32  BP230: 1:10 | Bullous pemphigoid | No new lesions appearing after **7 days**. | intravenous prednisolone | (20) |
| Yingjie Zhang et al. (2022) | 81 | M | NM | Hypertension | NM | inactivated COVID‐19 vaccine (Changchun Keygen Biological Products Co., Ltd., Changchun, China) | Third dose | Blisters with slight itching.  edematous dark erythema, blisters, blood blisters, and crusts | the entire body | 15 days | Subepidermal blister, inflammatory infiltrate in the blister and dermis adjacent to the blister,  BP 180 (1:32), and IgG and C3 | Bullous pemphigoid | Rapid improvement | intravenous prednisolone (60 mg/day) and gamma globulin (20 g/day) therapy | (20) |
| Mohammed Shanshal (2022) | 90 | F | NM | atrial fibrillation, hypertension, and primary biliary cirrhosis | long-term warfarin, bisoprolol, amlodipine, bendroflumethiazide, and ursodeoxycholic acid | Pfizer | First dose | itchy eczematous rash, left leg swelling.  widespread eczematous rash and excoriation marks | trunk and extremities, left leg | 1 week | subepidermal splitting with moderate subjacent inflammation.  C3.  anti-BMZ IgG autoantibodies | Dyshidrosiform Bullous Pemphigoid | NM | hydrocortisone 1% cream, moisturiser, oral antihistamine, and permethrin 5%. | (21) |
| Amanda dos Santos MD, Pallaci M. et al. | 79 | M | NM | Insulin-dependent type II diabetes, end-stage renal disease on hemodialysis, hypertension, and coronary artery disease | Insulin.  He denied any new medication. | Pfizer | First dose | diffuse firm and flaccid bullae | Started on right arm and then quickly became generalized. trunk, upper and lower extremities, head, face, feet but sparing the palms, soles, and mucous membranes. | 1 day | subepidermal blisters with eosinophils.  C3, IgG. | Bullous pemphigoid | The patient died of refractory septic shock secondary to pneumonia. | doxycycline, prednisone, niacinamide, hydroxyzine and topical triamcinolone.  IV dexamethasone.  rituximab infusion. | (22) |
| Timothy Cowan et al. | 71 | M | NM | type 2 diabetes  mellitus, an achilles tendon rupture and benign  prostatic hypertrophy. | metformin,  saxagliptin and tamsulosin.  No recent change in medication. | Vaxzevria Oxford-AstraZeneca | First dose | pruritic urticarial lesions | Arms, lateral edges of his feet, then spread over his entire body | 4 weeks | subepidermal blistering.  C3, IgG.  positive BP180 antibodies at a titre >5.00 and negative BP230 antibodies. | Bullous pemphigoid | After two weeks of  treatment, his BPDAI had reduced to an activity score  of 65 and damage score of 10 with extensive postinflammatory hyperpigmentation but minimal new  blisters. | gliptin was ceased. | (23) |
| Syeda S. Nida et al  2022 | 70 | M | NM | Parkinson’s disease, coronary artery disease, stage III chronic kidney disease, obstructive sleep apnea, chronic anemia, alcohol abuse disorder, renal cell carcinoma status post right radical nephrectomy, and squamous cell carcinoma of the lung in remission after chemoradiation | pimavanserin | Pfizer COVID-19 vaccine | second dose | painful pruritic maculopapular rash , tense bullae with negative Nikolsky sign | hands and extended proximally to trunk. | 2 days | Punch biopsy showed a sub-epidermal bulla with eosinophils  Direct immunofluorescence showed weak linear deposition of immunoglobulin G (IgG) and strong linear C3 deposition at the dermal-epidermal junction | bullous pemphigoid | NM | initiation of topical and systemic steroids (prednisone and clobetasol)  prednisone 60 mg for a total of one week and with full skin examinations every day to ensure no new blisters. After one week, prednisone was decreased to 40mg daily for one week, then 30mg daily for one week, then 20mg daily, then 10mg daily until his follow-up  topical clobetasol propionate 0.05% ointment for a total of two weeks. | (24) |
| Bryan Daines  2022 | 70 | M | NM | prostate and renal cancer (in remission) , hypertension and arthritis | amlodipine and naproxen | Pfizer-BioNTech (mRNA) | second dose | brightly erythematous, indurated plaques , intact and denuded bullae | on the trunk, arms, and legs , bilateral palms | 1 day | Lesional and perilesional biopsies were obtained for routine histopathology and direct immunofluorescence and revealed a subepidermal split with numerous eosinophils, and linear lgG and C3 staining along the basement membrane zone  serum ELISA testing revealed positive IgG BP 180 antibodies | Bullous pemphigoid | NM | Prednisone 40mg and triamcinolone 0.1% cream were initiated with an 80mg dose of intramuscular triamcinolone  limited further cyclosporin due to patient reaction  intravenous methylprednisolone (250mg daily × three days) and triamcinolone wet wraps and was discharged on oral methotrexate 15mg weekly, triamcinolone 0.1% ointment, and a prednisone taper starting at 60mg daily  Halobetasol 0.05% cream and mometasone 0.1% ointment | (25) |
| **Yukiko Nakahara**  2022 | 71 | M | NM | type 2 diabetes mellitus hemophagocytic lymphohistiocytosis (HLH) | dipeptidyl peptidase 4 in- hibitors (DPP4i) | Pfizer–BioNTech | second dose | multiple blisters without erythema  erythematous plaques with crust- ing lesions | on the neck and arms  over the face and sun-exposed areas of neck, palm, and fingers | After vaccination | A skin biopsy of the erythema on the neck revealed subepidermal cleft with a mixed inflammatory infiltrate of lymphocytes and eosinophils mostly localized in a superficial dermis  Direct immu- nofluorescence showed granular deposition of IgM and C3 at the basement membrane zone ,a typical his- tological picture of cutaneous LE. Indirect immunofluorescence (IIF) was negative. Anti-BP180 Ab was not detected | Bullous systemic lupus erythematosus | 4 weeks | Systemic corticosteroid, 20 mg of prednisone  Hydroxychloroquine 600 mg  Topical steroid | (26) |
| **Yin‐Cheng Chao, Kwei‐Lan Liu**  2023 | 49 | F | no | no | no | AstraZeneca | second dose | progressive pruritic annular confluent urticarial plaques  Tense vesicles and bullae with clear yellowish fluid | on the neck, trunk, and limbs | 3 day | biopsy showed a sub-epidermal blister and a perivascular infiltrate of eosinophils and lymphocytes in the dermis . Direct immunofluorescence test showed linear deposition of IgG and C3 at the dermal-epidermal junction. Indirect immunofluorescence assay revealed negative anti-intercellular substance antibody, but positive anti-basement membrane zone antibody | BP | NM | Intravenous methylprednisolone 80 mg/day and topical diflucortolone ointment twice a day  Azathioprine 50 mg/day was also prescribed in conjunction | (27) |
| Ze Guo et al  2022 | 67 | F | no | cerebral infarction | no | inactivated COVID-19 vaccine (Sinovac) | First dose | tense blisters over erythematous patches | on the trunk and extremities with rare mucosal involvement | 7 days | skin biopsy showed subepidermal detachment with eosinophils, neutrophils, and fibrin in the blister content, and a dermal inflammatory infiltrate  Indirect immunofluorescence (IIF) on monkey esophagus exhibited linear IgG deposition at the dermal-epidermal junction. In addition, direct immunofluorescence showed linear deposits of C3 along the basement membrane zone  Serum anti-BP180 autoantibodies were positive | BP | 2 weeks | systemic glucocorticoids, antibiotics, topical corticosteroids, and emollients | (28) |
| Ze Guo et al  2022 | 66 | F | no | cerebral infarction | no | inactivated COVID-19 vaccine (Sinovac) | First dose | tense blisters and erythematous patches | on the trunk and extremities accompanied by intense pruritus and rare mucosal involvement | 10 days | skin biopsy showed subepidermal detachment with eosinophils, neutrophils, and fibrin in the blister content and a dermal inflammatory infiltrate. Linear IgG deposition at the dermal-epidermal junction was seen on Indirect immunofluorescence. Again, direct immunofluorescence showed linear deposits of C3 along the BMZ  Serum anti-BP180 autoantibodies were positive | BP | 2 weeks | systemic glucocorticoids, antibiotics, topical corticosteroids, and emollients | (28) |
| Elif Afacan et al  2022 | 88 | F | NM | NM | NM | inactivated COVID-19 vaccine (Sinovac) | Second dose | NM | NM | 1 month | BP was confirmed with histopathology and direct immunofluorescence | BP | NM | TCS, OCS, MTX | (29) |
| Elif Afacan et al  2022 | 82 | F | NM | NM | NM | Pfizer–BioNTech | 3rd dose | NM | NM | 2 weeks | BP was confirmed with histopathology and direct immunofluorescence | BP | NM | TCS, OCS, Dapsone | (29) |
| Elif Afacan et al  2022 | 65 | M | NM | NM | NM | Pfizer–BioNTech | 3rd dose | NM | NM | 2 weeks | BP was confirmed with histopathology and direct immunofluorescence | BP | NM | TCS, DCN | (29) |
| Elif Afacan et al  2022 | 82 | F | NM | NM | NM | inactivated COVID-19 vaccine (Sinovac) | Second dose | NM | NM | 2 weeks | BP was confirmed with histopathology and direct immunofluorescence | BP | NM | TCS, OCS | (29) |
| Schmidt V. et al  2021 | 84 | F | NM | HTN, hyperthyreosis, osteoporosis and gastro-oesophageal reflux, lupus erythematosus, myocardial infarction, arteriosclerosis, hypercholesterolaemia, thrombophlebitis | NM | Moderna | 1st & 2nd | erythematous rash and eruption of blisters | arm, leg, and trunk | 4 week, 29 days | subepidermal blistering and subepidermal blistering and spongiosis with eosinophil accumulation and autoantibodies to BP 180 (1 : 320; normal range <1 : 80) and BP 230 (+++; normal range: 0; maximum range: ++++) in accordance with the diagnosis of BP. | BP | NM | NM | (30) |
| Fu Pa et al  2022 | 77 | M | No | Anemia | NM | Moderna | 2nd | ecchymoses at bilateral forearms, and legs and hemorrhagic blisters and papules | bilateral forearms, and legs | 3 week | sub-epidermal blistering with fibrin and abundant neutrophils//inear deposition of IgG and C3 at the dermis–epidermis junction | Bullous Pemphigoid & hemophilia A | NM | NM | (31) |
| Fabrizio Martora et al  2022 | NM | M | NM | NM | NM | Pfizer | NM | NM | NM | 15 days | NM | BP | NM | oral corticosteroids ± azathioprine | (32) |
| Altmann S et al | 70 | M | No | HTN, hyperlipidemia, and gastroesophageal reflux disease | NM | moderna | 2 dose | eruption, several large, geometric, eroded plaques and bullae with surrounding erythema | Trunk, and limbs | 1 week | subepidermal blister with eosinophils, linear deposition of IgG and C3 along the basement membrane zone | BP | persistent oral and cutaneous lesions at 2-week follow-up | combination of high-dose oral prednisone (1mg/kg/day), doxycycline 100mg twice daily, nicotinamide 500mg three times daily, and triamcinolone 0.1% cream, combined with gentamicin ointment under occlusion | (33) |
| Van W et al  2022 | 50 | F | NO | Asthma, HTN | monteleukast, rabeprazole, cetirizine, fluticasone/salmeterol, glycopyrronium bromide, | 2 dose of Pfizer  1 dose of moderna | 2^nd^ & 3rd | generalized pruritic rash | pink plaques with both non-hemorrhagic and hemorrhagic bullae scattered on the extremities, neck, chin, and torso her torso and extremities | 3 months | *linear deposition  *DIF showed a linear deposition of IgG and C3 along the dermal–epidermal junction | BP | her 16-week follow-up appointment, she was not developing any new bullae | a short tapering course of prednisone (from 50 to 5mg PO once daily) for 4weeks as methotrexate (15mg PO once weekly) was initiated | (34) |
| Van W et al  2022 | 82 | M | 2-month history of a pruritic rash | HTN and hypercholesterolemia | bisoprolol, rosuvastatin, acetylsalicylic acid (ASA), ramipril, and clopidogrel | pfizer | 1^st^ & 2nd | pruritic rash (non-hemorrhagic and hemorrhagic bullae were observed on the right forearm) | right forearm and legs | 10 days after first dose and 3 days after 2nd dose | subepidermal separation with the vesicle filled with serous fluid and a mixed infiltrate of eosinophils, neutrophils, and small lymphocytes. DIF showed a linear deposition for IgG and C3 along the dermal–epidermal junction with preferential staining of the roof of the split lymphocytes | BP | treated after 2 week | clobetasol propionate 0.05% ointment | (34) |
| Cowan TL et al  2023 | 82 | M | nm | nm | nm | AZ | nm | nm | Nm | 31 days | Nm | drug induced Bullous pemphigoid | nm | nm | (35) |
| Cowan TL et al  2023 | 62 | M | nm | nm | nm | pfizer | Nm | nm | nm | 123 days | Nm | drug induced Bullous pemfigoid | nm | nm | (35) |
| Cowan TL et al  2023 | 71 | M | nm | nm | nm | AZ | nm | nm | nm | 26 days | Nm | drug induced Bullous pemfigoid | nm | nm | (35) |
| Cowan TL et al  2023 | 60 | F | nm | nm | nm | AZ | nm | nm | nm | 5 days | Nm | drug induced Bullous pemfigoid | nm | nm | (35) |
| Baffa ME et al  2023 | 91 | F | No | NM | HTN, CKD | Pfizer | 2nd | severely pruritic rash and hemorrhagic blisters | trunk and limbs | 10 days | subepidermal detachment associated with an eosinophil-rich dermal inflammation. Direct immunofluorescence from the perilesional skin detected a linear deposition of IgG and complement 3 along the basement membrane zone (BMZ); indirect immunofluorescence using the salt-split-skin substrate showed linear deposition of circulating IgG along the epidermal-BMZ; ELISA demonstrated elevated IgG autoantibodies against BP180 NC16A | BP, and bullous herpes zoster | NM | oral prednisone at a starting dose of 0.5 mg/kg/day and topical clobetasol propionate 0.05% ointment once daily.And intravenous methylprednisolone (3 mg/kg/day) for 3 consecutive days. two doses of RTX 1000mg 2-week apart. Also, we proposed dupilumab as rescue therapy. A loading dose of 600mg sc followed by 300mg sc every 2weeks was thus administered | (36) |

1. Alshammari F, Abuzied Y, Korairi A, Alajlan M, Alzomia M, AlSheef M. Bullous pemphigoid after second dose of mRNA- (Pfizer-BioNTech) Covid-19 vaccine: A case report. Ann Med Surg (Lond). 2022;75:103420.

2. Khalid M, Lipka O, Becker C. Moderna COVID-19 vaccine induced skin rash. Vis J Emerg Med. 2021;25:101108.

3. Pérez-López I, Moyano-Bueno D, Ruiz-Villaverde R. Bullous pemphigoid and COVID-19 vaccine. Med Clin (Barc). 2021;157(10):e333-e4.

4. Larson V, Seidenberg R, Caplan A, Brinster NK, Meehan SA, Kim RH. Clinical and histopathological spectrum of delayed adverse cutaneous reactions following COVID-19 vaccination. J Cutan Pathol. 2022;49(1):34-41.

5. Agharbi FZ, Eljazouly M, Basri G, Faik M, Benkirane A, Albouzidi A, et al. Bullous pemphigoid induced by the AstraZeneca COVID-19 vaccine. Ann Dermatol Venereol. 2022;149(1):56-7.

6. Hung WK, Chi CC. Incident bullous pemphigoid in a psoriatic patient following mRNA-1273 SARS-CoV-2 vaccination. J Eur Acad Dermatol Venereol. 2022;36(6):e407-e9.

7. Bostan E, Yel B, Akdogan N, Gokoz O. New-onset bullous pemphigoid after inactivated Covid-19 vaccine: Synergistic effect of the Covid-19 vaccine and vildagliptin. Dermatol Ther. 2022;35(2):e15241.

8. Maronese CA, Di Zenzo G, Genovese G, Barei F, Monestier A, Pira A, et al. Reply to "New-onset bullous pemphigoid after inactivated Covid-19 vaccine: Synergistic effect of the Covid-19 vaccine and vildagliptin". Dermatol Ther. 2022;35(6):e15496.

9. Dell'Antonia M, Anedda S, Usai F, Atzori L, Ferreli C. Bullous pemphigoid triggered by COVID-19 vaccine: Rapid resolution with corticosteroid therapy. Dermatol Ther. 2022;35(1):e15208.

10. Hali F, Sr., Araqi L, Jr., Marnissi F, Meftah A, Chiheb S. Autoimmune Bullous Dermatosis Following COVID-19 Vaccination: A Series of Five Cases. Cureus. 2022;14(3):e23127.

11. Bardazzi F, Carpanese MA, Abbenante D, Filippi F, Sacchelli L, Loi C. New-onset bullous pemphigoid and flare of pre-existing bullous pemphigoid after the third dose of the COVID-19 vaccine. Dermatol Ther. 2022;35(7):e15555.

12. Shakoei S, Kalantari Y, Nasimi M, Tootoonchi N, Ansari MS, Razavi Z, et al. Cutaneous manifestations following COVID-19 vaccination: A report of 25 cases. Dermatol Ther. 2022;35(8):e15651.

13. Avallone G, Giordano S, Astrua C, Merli M, Senetta R, Conforti C, et al. Reply to 'The first dose of COVID-19 vaccine may trigger pemphigus and bullous pemphigoid flares: is the second dose therefore contraindicated?' by Damiani G et al. J Eur Acad Dermatol Venereol. 2022;36(6):e433-e5.

14. Pauluzzi M, Stinco G, Errichetti E. Bullous pemphigoid in a young male after COVID-19 mRNA vaccine: a report and brief literature review. J Eur Acad Dermatol Venereol. 2022;36(4):e257-e9.

15. Gambichler T, Hamdani N, Budde H, Sieme M, Skrygan M, Scholl L, et al. Bullous pemphigoid after SARS-CoV-2 vaccination: spike-protein-directed immunofluorescence confocal microscopy and T-cell-receptor studies. Br J Dermatol. 2022;186(4):728-31.

16. Russo R, Gasparini G, Cozzani E, D’Agostino F, Parodi A. Absolving COVID-19 Vaccination of Autoimmune Bullous Disease Onset. Front Immunol. 2022;13.

17. Young J, Mercieca L, Ceci M, Pisani D, Betts A, Boffa MJ. A case of bullous pemphigoid after the SARS-CoV-2 mRNA vaccine. J Eur Acad Dermatol Venereol. 2022;36(1):e13-e6.

18. Murryam S, Panagou E, Martyn-Simmons C. Bullous pemphigoid with subsequent milia enplaque following administration of the Pfizer–BioNTech COVID-19 vaccine. British Association of Dermatologists. 2022;186:ppe244–e58.

19. Nakamura K, Kosano M, Sakai Y, Saito N, Takazawa Y, Omodaka T, et al. Case of bullous pemphigoid following coronavirus disease 2019 vaccination. The Journal of dermatology. 2021;48(12):e606-e7.

20. Zhang Y, Lang X, Guo S, He H, Cui H. Bullous pemphigoid after inactivated COVID-19 vaccination: Case report. Dermatol Ther. 2022;35(8):e15595.

21. Shanshal M. Dyshidrosiform Bullous Pemphigoid Triggered by COVID-19 Vaccination. Cureus. 2022;14(6).

22. Amanda dos Santos M, Pallaci M. Bullous Pemphigoid Reaction After Second Dose of COVID-19 Vaccine.

23. COWAN T, WALLMAN L, MURRELL D. Severe bullous pemphigoid after Vaxzevria COVID-19 vaccination. Mucosa.5(1):27-9.

24. Nida SS, Tobon GJ, Wilson M, Chauhan K. A Patient Develops Bullous Rash After Receiving the Second Dose of COVID-19 Vaccine. Cureus. 2022;14(9):e29786.

25. Daines B, Madigan LM, Vitale PA, Khalighi M, Innes M. A new eruption of bullous pemphigoid following mRNA COVID-19 vaccination. Dermatology Online Journal. 2022;28(4).

26. Nakahara Y, Yamane M, Sunada M, Aoyama Y. SARS‐CoV‐2 vaccine‐triggered conversion from systemic lupus erythematosus (SLE) to bullous SLE and dipeptidyl peptidase 4 inhibitors‐associated bullous pemphigoid. The Journal of Dermatology. 2023;50(2):162-5.

27. Chao Y-C, Liu K-L. New-onset bullous pemphigoid triggered by AstraZeneca COVID-19 vaccine. Dermatologica Sinica. 2022;40(4):245.

28. Guo Z, Wang Y, Tang H, Fan M, Wang W, Ding Y, et al. Bullous Pemphigoid After Vaccination With the Inactivated Severe Acute Respiratory Syndrome Coronavirus 2 Vaccine: Two Cases in China. Wound Management & Prevention. 2022;68(11):22-5.

29. Afacan E, Edek YC, İlter N, Gülekon A. Can Covid‐19 vaccines cause or exacerbate bullous pemphigoid? A report of seven cases from one center. International journal of dermatology. 2022;61(5):626-7.

30. Schmidt V, Blum R, Möhrenschlager M. Biphasic bullous pemphigoid starting after first dose and boosted by second dose of mRNA-1273 vaccine in an 84-year-old female with polymorbidity and polypharmacy. Journal of the European Academy of Dermatology and Venereology: JEADV. 2021.

31. Fu P-A, Chen C-W, Hsu Y-T, Wei K-C, Lin P-C, Chen T-Y. A case of acquired hemophilia A and bullous pemphigoid following SARS-CoV-2 mRNA vaccination. Journal of the Formosan Medical Association. 2022;121(9):1872-6.

32. Martora F, Ruggiero A, Battista T, Fabbrocini G, Megna M. Bullous pemphigoid and COVID‐19 vaccination: Management and treatment reply to ‘Bullous pemphigoid in a young male after COVID‐19 mRNA vaccine: A report and brief literature review’by Pauluzzi et al. Journal of the European Academy of Dermatology and Venereology. 2022.

33. Altmann S, Jacobs D, Brown T, Kamath P, Krishnamurthy K. Treatment-Resistant Pemphigoid Following SARS-CoV-2 Vaccination. SKIN The Journal of Cutaneous Medicine. 2022;6(3):238-42.

34. Wan V, Chen D, Shiau CJ, Jung GW. Association between COVID-19 vaccination and bullous pemphigoid–a case series and literature review. SAGE Open Medical Case Reports. 2022;10:2050313X221131868.

35. Cowan TL, Huang C, Murrell DF. Autoimmune blistering skin diseases triggered by COVID-19 vaccinations: An Australian case series. Front Med. 2023;9:3959.

36. Baffa ME, Maglie R, Montefusco F, Pipitò C, Senatore S, Antiga E. Severe bullous pemphigoid following Covid‐19 vaccination resistant to rituximab and successfully treated with dupilumab. Journal of the European Academy of Dermatology and Venereology. 2022.
