# Supplementary material for: New-onset or flare-up of bullous pemphigoid associated with COVID-19 vaccines: a systematic review of case report and case series studies
Source: Front Med (Lausanne). 2024 Apr 8;11:1293920. doi: 10.3389/fmed.2024.1293920 (PMC11036870; doi:10.3389/fmed.2024.1293920)
Supplement: Supplementary file 3 [file Table_3.DOCX]

| **Authors (year)** | **Case age** | **Case gender** | **Patients' mucocutaneous disease history** | **clinical remission duration** | **Patients' other comorbidity** | **Drug history at the time of vaccination** | **Vaccine type** | **Vaccine dose** | **Description of mucocutaneous reactions** | **Distribution** | **Flair up onset** | **Skin or mucosal biopsy** | **Diagnosis** | **Resolution after (time)** | **Management of reactions** | **Ref** |
| --- | --- | --- | --- | --- | --- | --- | --- | --- | --- | --- | --- | --- | --- | --- | --- | --- |
| G Damiani et al. (2021) | 63 | F | bullous pemphigoid (BP) | 6 months | NM | oral prednisone | Moderna mRNA‐1273 vaccine | 1 | several blisters | trunk | 3 days | no | bullous pemphigoid flair | NM | oral prednisone | (1) |
| G Damiani et al. (2021) | 82 | F | bullous pemphigoid (BP) | 3 years | NM | oral prednisone and mycophenolate mofetil | Pfizer mRNABNT162b2 vaccine | first dose | small blisters | arms and legs | 3 days | no | bullous pemphigoid flair | NM | Oral prednisone | (1) |
| G Damiani et al. (2021) | 84 | M | bullous pemphigoid (BP) | 4 years | NM | oral prednisone and azathioprine | Moderna mRNA‐1273 vaccine | first dose | mild blistering lesions | trunk | 2 weeks | no | bullous pemphigoid flair | NM | Oral prednisone | (1) |
| Federico Bardazzi et al. (2022) | 57 | F | bullous pemphigoid (BP) | NM | NM | NM | Moderna | Third dose | pruritic and bullous skin eruption | Trunk, arms | 7 days | ELISA: BP180 (10 U/ml)  BP320 (3 U/ml) | bullous pemphigoid flare | NM | Topical high potency steroids twice daily for 15 days, then once daily until resolution | (2) |
| Federico Bardazzi et al. (2022) | 62 | M | bullous pemphigoid (BP) | NM | NM | NM | Pfizer | Third dose | pruritic and bullous skin eruption | Trunk, arms | 7 days | ELISA: BP180 (104 U/ml)  BP320 (26 U/ml) | bullous pemphigoid flare | NM | Topical high potency steroids twice daily for 15 days, then once daily until resolution | (2) |
| Elena Calabria et al. (2022) | 74 | F | bullous pemphigoid (BP) | NM | NM | NM | CoronaVac | First dose | NM | NM | 1 week | hematoxylin and eosin: +  direct immunofluorescence histology: + | bullous pemphigoid flare | NM | Doxycycline, topical corticosteroid,  oral corticosteroid | (3) |
| Elena Calabria et al. (2022) | 65 | F | bullous pemphigoid (BP) | NM | NM | NM | CoronaVac | Second dose | NM | NM | 1 week | hematoxylin and eosin: +  direct immunofluorescence histology: + | Bullous pemphigoid flare | NM | topical corticosteroid,  MTX | (3) |
| Elena Calabria et al. (2022) | 71 | M | bullous pemphigoid (BP) | NM | NM | NM | CoronaVac | Second dose | NM | NM | 1.5 month | hematoxylin and eosin: +  direct immunofluorescence histology: + | bullous pemphigoid flare | NM | Azathioprine. topical corticosteroid,  oral corticosteroid | (3) |
| Mary M Tomayko et al. (2021) | 83 | M | bullous pemphigoid (BP) | NM | NM | NM | Pfizer | First dose | NM | NM | 7 days | H&E staining: nd  DIF at DEJ : nd BP180/230: nd | bullous pemphigoid flare | Ongoing at d 45 | OCS, TCS | (4) |
| Juay Y. et al. (2021) | 70 | F | bullous pemphigoid (BP) | NM | NM | prednisolone | Tozinameran (Pfizer) | First dose | NM | NM | 2 weeks | NM | bullous pemphigoid flare | NM | Prednisolone, clobetasol | (5) |
| Michiel Happaerts et al  2022 | 75 | M | BP | 1 years | chronic kidney disease stage 3b, arterial hypertension, and insulin-dependent diabetes mellitus, polyneuropathy | unclear | AstraZeneca | First dose | mul- tiple hematomas, hemorrhagic BP | right arm and left buttock | 10 days | no | Acquired hemophilia A with a relapse of bullous pemphigoid | NM | recombinant activated factor VII , emicizumab 270 mg weekly , rituximab 375 mg/m2 weekly , methylprednisolone 64 mg daily | (6) |
| Elif Afacan et al  2022 | 74 | F | BP | 5 months | NM | NM | inactivated COVID-19 vaccine (Sinovac) | First dose | NM | abdomen and upper thighs, lateral thigh | 1 week | BP was confirmed with histopathology and direct immunofluorescence | experienced a flare of previous BP | NM | TCS, DCN, OCS, MTX | (7) |
| Elif Afacan et al  2022 | 65 | F | BP | 2 years | NM | NM | inactivated COVID-19 vaccine (Sinovac) | 2^nd^ dose | NM | NM | 1 week | BP was confirmed with histopathology and direct immunofluorescence | experienced a flare of previous BP | NM | TCS, MTX | (7) |
| Elif Afacan et al  2022 | 71 | M | BP | 6 months | NM | NM | inactivated COVID-19 vaccine (Sinovac) | 2^nd^ dose | NM | NM | 1.5 month | BP was confirmed with histopathology and direct immunofluorescence | experienced a flare of previous BP | NM | TCS,OCS,AZA | (7) |
| Cowan TL et al  2023 | 82 | M | BP | NM | NM | NM | AZ | NM | NM | NM | 92 days | NM | BP flair | NM | NM | (8) |
| Cowan TL et al  2023 | 83 | M | BP | NM | NM | NM | pfizer | NM | NM | NM | 90 days | NM | BP flair | NM | NM | (8) |
| Cowan TL et al  2023 | 86 | F | BP | NM | NM |  | pfizer | NM | NM | NM | 21 days | NM | BP flair | NM | NM | (8) |

1. Damiani G, Pacifico A, Pelloni F, Iorizzo M. The first dose of COVID-19 vaccine may trigger pemphigus and bullous pemphigoid flares: is the second dose therefore contraindicated? J Eur Acad Dermatol Venereol. 2021;35(10):e645-e7.

2. Bardazzi F, Carpanese MA, Abbenante D, Filippi F, Sacchelli L, Loi C. New-onset bullous pemphigoid and flare of pre-existing bullous pemphigoid after the third dose of the COVID-19 vaccine. Dermatol Ther. 2022;35(7):e15555.

3. Calabria E, Canfora F, Mascolo M, Varricchio S, Mignogna MD, Adamo D. Autoimmune mucocutaneous blistering diseases after SARS-Cov-2 vaccination: A Case report of Pemphigus Vulgaris and a literature review. Pathol Res Pract. 2022;232:153834.

4. Tomayko MM, Damsky W, Fathy R, McMahon DE, Turner N, Valentin MN, et al. Subepidermal blistering eruptions, including bullous pemphigoid, following COVID-19 vaccination. J Allergy Clin Immunol. 2021;148(3):750-1.

5. Juay L, Chandran NS. Three cases of vesiculobullous non-IgE-mediated cutaneous reactions to tozinameran (Pfizer-BioNTech COVID-19 vaccine). J Eur Acad Dermatol Venereol. 2021;35(12):e855-e7.

6. Happaerts M, Vanassche T. Acquired hemophilia following COVID‐19 vaccination: Case report and review of literature. Research and Practice in Thrombosis and Haemostasis. 2022;6(6):e12785.

7. Afacan E, Edek YC, İlter N, Gülekon A. Can Covid‐19 vaccines cause or exacerbate bullous pemphigoid? A report of seven cases from one center. International journal of dermatology. 2022;61(5):626-7.

8. Cowan TL, Huang C, Murrell DF. Autoimmune blistering skin diseases triggered by COVID-19 vaccinations: An Australian case series. Front Med. 2023;9:3959.
